# Combined effect of chitosan coating and modified atmosphere packaging on fresh‐cut cucumber

**DOI:** 10.1002/fsn3.937

**Published:** 2019-02-06

**Authors:** Ibukunoluwa F. Olawuyi, Jong Jin Park, Jae Jun Lee, Won Young Lee

**Affiliations:** ^1^ School of Food Science and Biotechnology Kyungpook National University Daegu Korea

**Keywords:** argon‐based, chitosan, fresh‐cut cucumber, MA packaging, nitrogen‐based, shelf life

## Abstract

Fresh‐cut cucumber was coated with different concentrations of edible chitosan solutions and packaged in air‐, nitrogen‐, and argon‐based MA to preserve quality and extend shelf life. The effectiveness of individual and combined treatments on some quality parameters was examined at intervals during 12 days storage at a temperature of 5°C. The concentration of chitosan solutions significantly affected the performance of fresh‐cut cucumber in MA packages. Improved quality retention and reduced carbon dioxide production were observed in chitosan‐coated fresh‐cut samples. Argon‐based MA packaged samples exhibited better potential than air and nitrogen‐based MA packaging in retarding tissue respiration, physiological changes, chlorophyll degradation, and extending shelf life of fresh‐cut cucumber. Combined chitosan coating with MA packaging maintained quality, microbial safety, and extended the shelf life of fresh‐cut cucumber.

## 1 INTRODUCTION

Fresh‐cut products are minimally processed fruits or vegetables that have been trimmed, peeled, and/or cut into a ready‐to‐eat product, which is subsequently packaged to offer consumers high nutrition, convenience, and flavor while maintaining freshness (IFPA, 2004; Rico, Martin‐Diana, Barat, & Barry‐Ryan, 2007). Recently, consumers are increasingly demanding for fresh‐cut fruits and vegetables due to the convenience it offers (Rico et al., 2007). Fresh‐cut tropical fruits on the market today include apple, melons, cantaloupe, banana, watermelon, mangoes, mangosteen, rambutan, jackfruit, pummelo, papaya, durian, grapefruit, pineapples, and fruit mixes (Sidhu & Al‐Zenki, 2005). Growing consumer's interest has led researchers into developing fresh‐cut from other fruits and vegetables such as broccoli, eggplant, and cucumber among others.

Cucumber, which is largely consumed as fresh fruit, vegetable in salads, or pickled product, is attracting more interest as a minimal processed fruit. Processing into fresh‐cut may be another way to improve its consumption and reduce postharvest losses by reducing quantity loss during storage of whole cucumber fruits. Subsequently, fresh‐cut fruits are even more perishable and have a very short shelf life due to minimal processing which directly affects fruit physiology as cutting increases tissue respiration rates (Artés & Allende, 2005). Minimum processing reduces fruit tissue integrity, triggering deterioration processes such as browning and softening, water loss as well as production of off‐flavors (Gil & Allende, 2012). The removal of the natural protective barrier of epidermis, increased humidity and solute leakage on fruit surfaces, provides optimum conditions for the growth of microorganisms (Oms‐Oliu, Soliva‐Fortuny, & Martín‐Belloso, 2008). These physiological defects accompanied by minimally processed fresh‐cut fruits have stimulated research on new ways to extend their shelf life and preserve quality (Coppens d'Eeckenbrugge, Leal, Bartholomew, Paull, & Rohrbach, 2003).

A recent approach to prolong the shelf life of fresh‐cut fruit and vegetables is the use of edible coatings either alone or combined with modified atmosphere packaging. Numerous studies on improving the shelf life of fresh‐cut fruits are extensive with the application of modified atmosphere packaging (Bai, Saftner, Watada, & Lee, 2001; Farber et al., 2003; Oms‐Oliu, Soliva‐Fortuny, & Martín‐Belloso, 2007), and edible coatings and films (Min & Krochta, 2005; Olivas & Barbosa‐Cánovas, 2005; Rico et al., 2007; Rojas‐Graü, Tapia, Rodríguez, Carmona, & Martin‐Belloso, 2007).

Edible coatings are material used for wrapping foods to extend their shelf life of the product, which may be consumed together with food with or without further removal (Pavlath & Orts, 2009). Chitosan, a polysaccharides‐based edible coating, has been successfully used to coat fresh‐cut fruits (Ali, Noh, & Mustafa, 2015; Bal, 2018; Chien, Sheu, & Yang, 2007; Jianglian & Shaoying, 2013).

MA packaging technology has been representatively used to maintain quality of fresh‐cut products during preservation by carbon dioxide elevation and reduction in oxygen levels (Saltveit, 2001; Singh, Hedayetullah, Zaman, & Meher, 2014; Zhang, Quantick, Grigor, Wiktorowicz, & Irven, 2001). In recent years, inert gas‐based food preservation technology has been successfully used to preserve freshness of fresh‐cut fruits and vegetables (Zhang et al., 2001). When inert gases such as argon are dissolved in water under certain pressure, water molecules form a cage of polyhedral structure that can hold inert gas molecules, known as the clathrate hydrate (Linga, Kumar, & Englezos, 2007; Yang et al., 2010). Fortunately, these gases are chemically stable and they do not have an adverse effect in human (Wu, Zhang, & Wang, 2012; Zhang et al., 2001).

Argon, as an alternative to nitrogen, has been approved to be used in European Union because of the properties of being odorless, inert, and tasteless (Day, 2007; Greenwood & Earnshaw, 2012). Argon is denser than nitrogen, (being 1.43 times denser) and thus it flows in a laminar type, like a liquid, through air space, unlike nitrogen (Spencer, 1995). Argon has been reported for its effectiveness in controlling respiratory rate and excludes oxygen more efficiently than nitrogen (Spencer, 1995). Argon as a main component of the air has also been reported to decrease microbial growth and improve the quality of products like broccoli and lettuce (Day, 1996; Day, 1998; Jamie & Saltveit, 2002; Zhang, Zhan, Wang, & Tang, 2008).

Many reports have been published on minimally processed fruits and vegetable; however, to the best of our knowledge, there has not been any published information on the effect of chitosan coating and nitrogen/argon‐based MA packaging on fresh‐cut cucumber. Therefore, this research focused on the effects of chitosan and nitrogen/argon‐based MA packaging on the quality and shelf life of fresh‐cut cucumber.

## 2 MATERIALS AND METHODS

### 2.1 Fruits and other materials

Freshly harvested cucumber (about 9 weeks after planting) was purchased directly from a local farm in Gwangju, Korea and stored in the refrigerator at 5°C for 24 hr before experimenting. The fruits were further sorted base on size, greenness, and free from blemish. Chitosan powder (Chitosan powder 100%, Symbiosal Biotech. Co. Ltd, Mokpo, Korea) of low molecular weight (10.5 cps, 20°C) was used in this study to aid easy solubility in solvent. Analytical grade acetic acid was purchased from Daejung Chemical Co. Ltd, Gyeonggi‐do, Korea. Composite films of polypropylene + polyamide (20 × 30 cm) were used in this study because of its excellent gas permeability properties. Airzero nozzle type vacuum and gas flushing machine (AZ‐450E) was used for MA packaging. Gas was mixed and supplied by Daedong Airgen, Daegu, Korea. Gas composition in each chamber include Air chamber (79% nitrogen, 20.9% oxygen, 0.03% carbon dioxide), Nitrogen‐base (79% nitrogen, 3% oxygen, 18% carbon dioxide), Argon‐base (79% argon, 3% oxygen, 18% carbon dioxide) (2.6%:17.9%) approximately.

### 2.2 Preparation of coating solution and experimental design

A preliminary study was conducted to select suitable storage temperature (0, 5, 10, 15°C) and to determine the average shelf life of fresh‐cut cucumber. Storage at 5°C preserved fresh‐cut cucumber for about 4 days; hence, this condition was selected for this study. Cucumber was washed in tap water and sliced into about 10 mm thickness. Two chitosan coating solutions were prepared and distilled water was used as control (0% chitosan). Chitosan solutions were prepared using the method described by Ali et al. (2015). To prepare coating concentrations, 1.0% chitosan concentration (10 g/L) and 2% concentration (20 g/L) chitosan powder, each was dissolved in distilled water (60°C) containing 10 ml of glacial acetic acid with the aid of magnetic stirrer for 5 hr. The pH of the solution was adjusted to 5.6–5.9, by 1 N NaOH. Cucumber slices were then divided into three portions based on dipping solutions. Single layer coating was carried out in batches by completely submerging 500 g sliced cucumber in 1 L solution for 2 min and then coated samples are allowed to dry inside a laminar airflow cabinet for about 30 min. Finally, 100 g coated fresh‐cut cucumber were packed into each packaging film, gas‐filled and sealed using the automated MA packaging machine. For each treatment, three packages (triplicate) were stored at 5°C, 60% RH in the refrigerator (Samsung, CRF‐1764D). Samples were analyzed at 3 days interval for 12 days.

### 2.3 Headspace gas analysis

The headspace gas compositions of packaged samples were analyzed with a digital gas analyzer (Quantek Gas Analyzer Model 902D, USA). Gas concentration was measured by penetrating the needle probe of the device into the package film containing the sample. Values carbon dioxide was recorded from display screen located on the instrument and result calculated as % carbon dioxide using the following equation:

$${\text{CO}}_{2}\;\text{produced}\;\left(\%\right)={\text{CO}}_{2\mathrm{fin}}-{\text{CO}}_{2\mathrm{in}}$$

where CO_2in_ is the carbon dioxide concentration of first day, while CO_2fin_ is the carbon dioxide on the final day of storage.

### 2.4 Weight loss and texture (firmness)

Weight of whole package on each day of analysis was measured by using laboratory scale digital weighing balance (Mettler Toledo, CH/PL 3002). Weight losses were determined comparing weight of samples (initial weight, 100 ± 3 g) before and after the storage period. The values were expressed as weight loss percentage with regard to the initial weight.

$$\text{Weight loss}\;\left(\%\right)=\frac{\left(W_{\mathrm{in}}-W_{\mathrm{fin}}\right)}{W_{\mathrm{in}}}\times 100$$

where *W*_in_ is the weight of the first day and *W*_fin_ the weight of final day.

Firmness was measured as the maximum force (g) required to puncture the flesh of fresh‐cut cucumber. A penetration test was conducted on the surface of fresh‐cut cucumber using a texture analyzer (Compac‐100, Scientific Co., Tokyo, Japan) with a 5 mm diameter cylindrical probe. Samples were penetrated to a depth of 12 mm. The speed of the probe was 3 mm/s per each penetration. Measurements were carried out in quadruplicate on each packaged sample.

### 2.5 Color values, yellowing index and whiteness index

Surface color of fresh‐cut cucumber was measured with a chroma meter (CR‐300, Minolta Co., Osaka, Japan) using L* (lightness), a* (red‐green), and b* (yellow‐blue) values. The color was expressed as yellowing index (Kochhar & Kumar, 2015) of peel and whiteness index (Amanatidou, Slump, Gorris, & Smid, 2000) of fresh‐cut surface. The value of Δ*E* defines the color difference and is calculated by follows:

$$\text{Yellowing index (YI)}=142.48\times \frac{b}{L}$$

$$\text{Whiteness Index (WI)}=100-{\left[{\left(100-L\right)}^{2}+\left(a^{2}+b^{2}\right)\right]}^{1/2}$$

### 2.6 Chlorophyll content

The chlorophyll contents of fresh‐cut cucumbers were determined according to the method proposed by Zhang et al. (2008). Fresh‐cut cucumbers (5 g) were homogenized with 20 ml of 80% acetone in a tissue homogenizer (Daihan Sci. Co., HG‐15A, Korea) at 2,000 *g* (relative centrifugal force) for 30 s. The mixture was filtered using vacuum filtering and finally centrifuged at 2,000 *g* (relative centrifugal force) for 15 min. The absorbance of the filtered homogenate was measured at 647.0 and 664.5 nm on a UV spectrophotometer (Shimazdu Co. UV‐2550, Tokyo, Japan). The chlorophyll content was calculated using the following equation:

$$\text{Chlorophyll}\;\left(\text{mg/g}\right)=17.95A_{647}+7.9A_{664.5}$$

### 2.7 Microbiological analysis

All samples were analyzed for total bacteria and fungi (yeast and molds) counts. Ten grams of fresh‐cut cucumber were aseptically packed using sample bag (190 × 300 mm, 3M Co., MN, USA) and diluted with 90 ml of 0.1% peptone water. The samples were homogenized by a stomacher (SH‐001, Shimskyu, Tokyo, Japan) at high speed for 3 min. The bacteria were determined on plate count agar (PCA; Becton Dickinson, NJ, USA) following incubation at 37°C for 48 hr and represented as log CFU/g for bacteria. Fungi were counted on potato dextrose agar (PDA; Becton Dickinson, NJ, USA). The incubation temperature was 25°C and plates were examined after 48 hr.

### 2.8 Statistical analysis

For statistical analysis, all experiments were carried out in a minimum of triplicates and data were processed by analysis of variance and mean separated by Duncan's multiple range tests (*p* < 0.05) using by SPSS software (Version 20, SPSS lnc. Chicago, IL, USA).

## 3 RESULTS AND DISCUSSION

### 3.1 Headspace gas analysis

**Figure 1:**
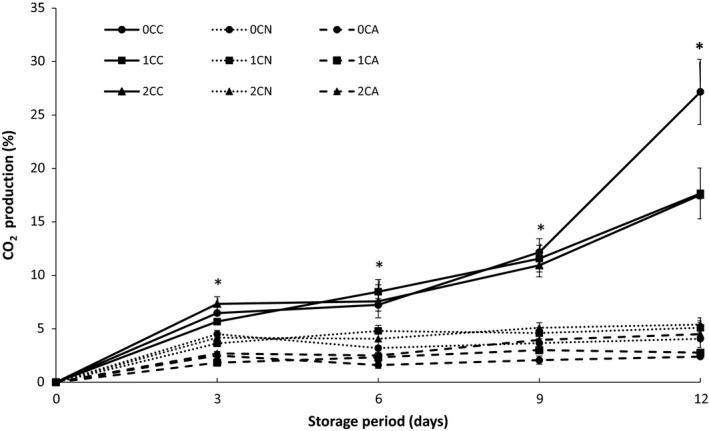
CO_2_ production of fresh‐cut cucumber stored at 5°C for 12 days. Where 0C, 1C, and 2C represents 0%, 1%, and 2% chitosan concentration respectively, and C, N, and A represents control (air‐based), N (nitrogen‐based), and A (argon‐based) MAP respectively. Values represent the mean ± SD of three determinations. “*” Indicates significant differences (*p* < 0.05) between treatments at the same storage day

Respiration rate is one of the major factors contributing to postharvest losses of fruit (Bhande, Ravindra, & Goswami, 2008).The consumption of oxygen and subsequent carbon dioxide production in MA packages is a factor of tissue respiration occurring in the fresh‐cut product (Ghidelli, Mateos, Rojas‐Argudo, & Pérez‐Gago, 2014). Results obtained in this study showed a high production of carbon dioxide (27.17%) in uncoated and air‐packaged samples (Figure 1). As seen in Figure 1, nitrogen‐ and argon‐based MA packaged samples showed significant differences from air‐packaged samples throughout the storage period. However, 1% and 2% coated air‐packaged samples (1CC and 2CC) did not show significant differences with uncoated sample (0CC) till 9 days. However, apparent differences were observed after 12 days of storage. Using the rate of carbon dioxide production, this result confirms chitosan coating has gaseous barrier properties. Several studies have described the beneficial effects of polysaccharide edible coatings on reducing respiration rate of fresh‐cut products, which is due to their good gaseous barrier (Ghidelli et al., 2014; Olivas & Barbosa‐Cánovas, 2005). Wong, Tillin, Hudson, and Pavlath (1994) also reported a lower carbon dioxide production rate (50%–70% reduction) in apple pieces coated with several polysaccharide‐based coatings. Nitrogen‐ and argon‐based MA packages had reduced respiration (very low carbon dioxide production) which delayed physiological changes caused by respiration during storage period. In contrast, combined application of chitosan coating with nitrogen or argon MA packaging significantly influenced consumption of oxygen, and subsequently, the reduced production of carbon dioxide maintained a low respiration rate which is an important factor in metabolic reactions and deterioration of fruits during storage.

### 3.2 Weight loss and texture (firmness)

**Figure 2:**
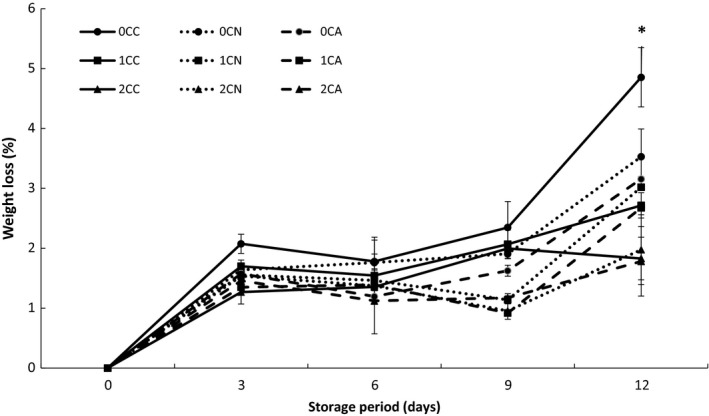
Percentage loss in weight of fresh‐cut cucumber stored at 5°C for 12 days. Where 0C, 1C, and 2C represents 0%, 1%, and 2% chitosan concentration respectively, and C, N, and A represents control (air‐based), N (nitrogen‐based), and A (argon‐based) MAP, respectively. Values represent the mean ± SD of three determinations. “*” Indicates significant differences (*p* < 0.05) between treatments at the same storage day

Fresh‐cut fruits are more prone to weight loss which makes it an essential parameter to be evaluated during storage experiment. As shown in the result Figure 2, all samples experienced increased weight loss throughout the storage period. However, the percentage weight loss differs by each treatment with uncoated air‐packaged sample having the highest loss in weight with a value of 4.85%, while 2% chitosan‐coated and argon‐based MA packaged samples had the least weight loss of 1.78% after 12 days of storage. Weight loss in the fresh‐cut fruit is due to moisture loss from the surface of the fresh‐cut which may cause some physiological changes. The concentration of chitosan coating was observed to affect weight loss, but no obvious effect of MA gaseous composition was observed in the result from this study (Figure 2). Previous studies have described the beneficial effects of edible coatings in reducing weight loss of minimally processed fruit and vegetables (Chien et al., 2007). This beneficial effect of chitosan coating may be due to the barrier created by chitosan polymers used, which in turn reduced gas exchange and water loss from fresh‐cut samples (Brasil, Gomes, Puerta‐Gomez, Castell‐Perez, & Moreira, 2012; Brecht, 1995).

**Figure 3:**
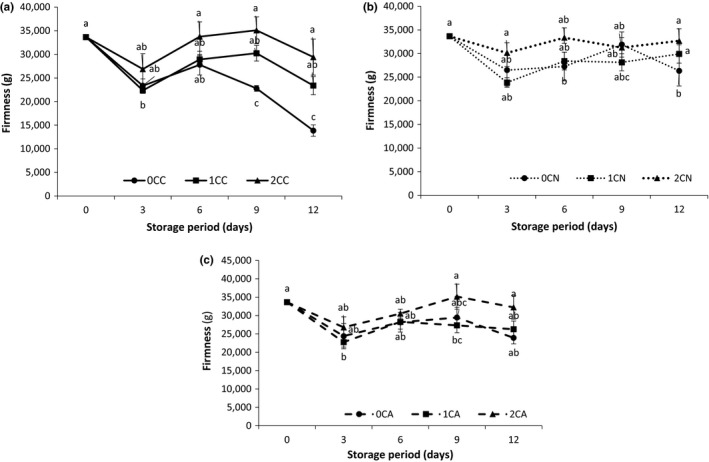
Texture (a), effect of chitosan coating on firmness of air‐based (b), effect of chitosan coating on firmness of nitrogen‐based (c), effect of chitosan coating on firmness of argon‐based MA packaged fresh‐cut cucumber. Where 0C, 1C, and 2C represents 0%, 1%, and 2% chitosan concentration, respectively, and C, N, and A represents control (air‐based), N (nitrogen‐based), and A (argon‐based) MAP, respectively. Values represent the mean ± SD of three determinations. Different alphabets are significantly different (*p* < 0.05) between treatments at the same storage day

Firmness is another important factor for quality of fresh‐cut fruit (Liu et al., 2014).The firmness of all samples experienced a dramatic decrease after the 3 days of storage (Figure 3a–c). This decrease may be attributed to the initial rapid loss in moisture as shown in Figure 2a. At the end of storage, it was observed that chitosan coating helped to preserve the firmness of fresh‐cut cucumber. The order of firmness retention was 0% ˂ 1% ˂ 2% chitosan concentration in all fresh‐cut cucumber packages, with 2% chitosan‐coated samples having the highest values for firmness overall. MA packaging complemented chitosan coating in maintaining the firmness of the fresh‐cut product. Meng, Zhang, Zhan, and Adhikari (2014) also reported that the application of pressurized Ar can delay the softening of texture in cucumbers during storage. Low oxygen and elevated carbon dioxide concentration in the nitrogen/argon‐based MA packages reduces enzymatic activity of tissue degrading enzymes (e.g., pectinase) which are related to fruit softening (Brasil et al., 2012; Martiñon, Moreira, Castell‐Perez, & Gomes, 2014).

### 3.3 Color values; yellowing index and whiteness index

**Figure 4:**
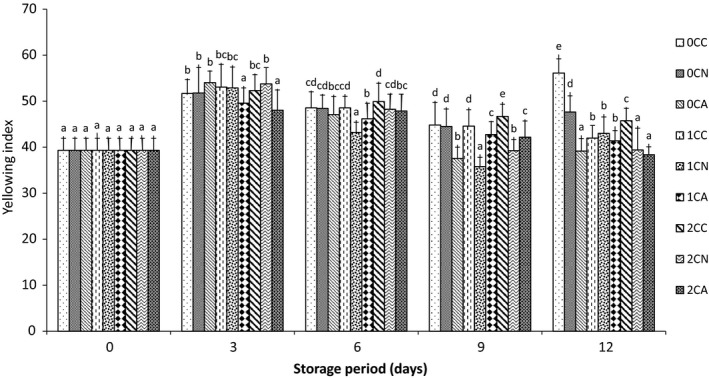
Yellowing index of fresh‐cut cucumber peel stored at 5°C for 12 days. Where 0C, 1C, and 2C represents 0%, 1%, and 2% chitosan concentration, respectively, and C, N, and A represents control (air‐based), N (nitrogen‐based), and A (argon‐based) MAP, respectively. Values represent the mean ± SD of three determinations. Different alphabets are significantly different (*p* < 0.05) between treatments at the same storage day

Color is an important quality parameter that affects consumers’ acceptance of fresh‐cut products. In this study, the yellowing index of peel and whiteness index of surface of fresh‐cut were used to quantify the effects of various treatments on the quality of fresh‐cut cucumber samples. Fruits remain living, respiring tissues after harvest. In freshly harvested green fruits, yellow carotenoids coexist with green chlorophylls. Since cucumber is a non‐climacteric green fruit, it produces very low ethylene. However, during senescence chlorophyll is rapidly degraded, exposing the lighter yellow pigments. Within few days of storage, most green vegetables will undergo unmasking of chlorophyll losing their dark green color and begin to turn yellow (Kanellis, Morris, & Saltveit, 1986; Moalemiyan & Ramaswamy, 2012). From the result in Figure 4, the yellowing index of all samples increased, indicating a general depletion of green color of the cucumber peel. About 1% and 2% chitosan‐coated samples were observed to have lower values for yellowing index in all the MA packages. Overall, the peel of uncoated air‐packaged samples had the highest yellowing index with 56.12 and the least value of 38.36 was in 2% chitosan‐coated and argon‐based MA packaged samples. This result depicts that chitosan coating reduced yellowing in cucumber by retarding degradation of chlorophyll. Additional reductions in yellowing of cucumber peel were observed in nitrogen‐ and argon‐based MA packaged samples. This may be due to the elevated CO_2_ concentration in both nitrogen‐ and argon‐based packaged samples. This assumption is in agreement with the result reported by Bastrash, Makhlouf, Castaigne, and Willemot (1993) wherein low oxygen and high carbon dioxide storage atmosphere inhibited yellowing of cut broccoli at low temperatures.

**Figure 5:**
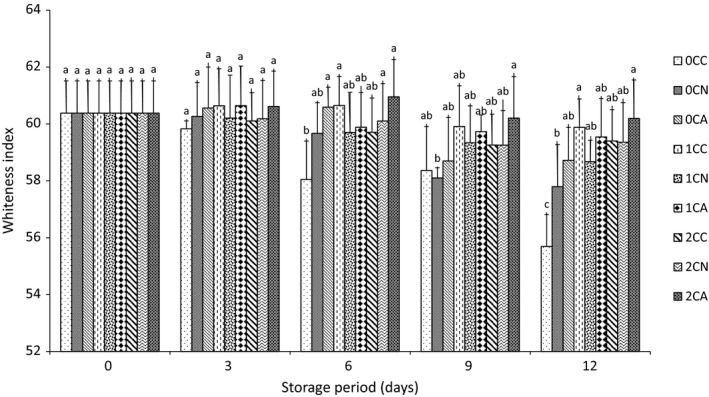
Whiteness index of fresh‐cut cucumber surface stored at 5°C for 12 days. Where 0C, 1C, and 2C represents 0%, 1%, and 2% chitosan concentration, respectively,and C, N, and A represents control (air‐based), N (nitrogen‐based), and A (argon‐based) MAP, respectively. Values represent the mean ± SD of three determinations. Different alphabets are significantly different (*p* < 0.05) between treatments at the same storage day

Similar trend was also observed in the result of the whiteness of the fresh‐cut surface (Figure 5). Chitosan coating and nitrogen/argon‐based MA packaging preserved the whiteness of the samples by reducing enzymatic browning that causes unpleasant color change. At the end of storage, all chitosan‐coated fresh‐cut samples showed better whiteness value than uncoated samples. Similar results have also been reported by Ghidelli et al. (2014) for fresh‐cut eggplant.

### 3.4 Chlorophyll content

Chlorophyll is mainly responsible for green color of cucumber peel. It plays vital role in the appearance and acceptance of cucumber. As the green pigment in cucumbers, when chlorophyll is decomposed, it leads to the development of yellowness that reduces their sensory appeal and market value (Barrett, Beaulieu, & Shewfelt, 2010). The result showed that chlorophyll was significantly (*p* < 0.05) affected by both chitosan coating and MA packaging (Table 1). Chlorophyll content of fresh‐cut cucumber decreased during storage irrespective of the treatment combination. Similar trend was observed in the result of yellowing index (Figure 4). After storage period, uncoated and air‐packaged fresh‐cut samples had the highest chlorophyll degradation with the lowest total chlorophyll content of 1.71 mg/g, while 2% chitosan‐coated and argon‐based MA packaged samples retained more chlorophyll with the highest value of 3.07 mg/g. Throughout storage, argon‐based MA packaged samples had consistently higher chlorophyll content than both nitrogen‐ and air‐packaged fresh‐cut samples. Argon has the potential of minimizing or lowering color change (Meng et al., 2014). This might be due to the fact that argon could be capable of slowing down the conversion of chlorophyll to yellow‐olive‐colored pheophytin in the cucumber flesh (Krebbers, Matser, Koets, & Van den Berg, 2002). It is also noteworthy to report that either chitosan coating or argon MA packaging can significantly reduce chlorophyll degradation; however, their combinations were observed to be more effective in this study.

**Table 1:** Chlorophyll content of fresh‐cut cucumber stored at 5°C for 12 days

Chlorophyll content (mg/g)	Storage period (days)				
Treatment	0	3	6	9	12
0CC	5.43 ± 0.00^a^	4.06 ± 0.02^d^	3.58 ± 0.04^d^	2.80 ± 0.23^d^	1.71 ± 0.18^e^
0CN	5.43 ± 0.00^a^	4.48 ± 0.18 ^cd^	3.11 ± 0.09^e^	2.35 ± 0.12^e^	1.97 ± 0.09^d^
0CA	5.43 ± 0.00^a^	4.66 ± 0.03^c^	4.05 ± 0.30^c^	2.96 ± 0.02^d^	2.08 ± 0.09^d^
1CC	5.43 ± 0.00^a^	4.70 ± 0.32^c^	4.01 ± 0.04^c^	3.52 ± 0.04^c^	2.77 ± 0.10^b^
1CN	5.43 ± 0.00^a^	4.68 ± 0.14^c^	3.96 ± 0.10^c^	2.99 ± 0.07^d^	2.42 ± 0.10^c^
1CA	5.43 ± 0.00^a^	5.01 ± 0.13^b^	4.23 ± 0.43^b^	3.32 ± 0.10^c^	2.95 ± 0.16^ab^
2CC	5.43 ± 0.00^a^	4.89 ± 0.02^bc^	4.60 ± 0.21^ab^	3.98 ± 0.04^b^	2.97 ± 0.04^ab^
2CN	5.43 ± 0.00^a^	4.94 ± 0.07^b^	4.42 ± 0.03^ab^	3.80 ± 0.06^b^	2.90 ± 0.09^ab^
2CA	5.43 ± 0.00^a^	5.36 ± 0.01^a^	4.72 ± 0.24^a^	4.21 ± 0.20^a^	3.07 ± 0.06^a^

*Note*

Where 0C, 1C, and 2C represents 0%, 1%, and 2% chitosan concentration respectively, and C, N, and A represents control (air‐based), N (nitrogen‐based), and A (argon‐based) MAP, respectively. Values in the same column with different superscripts are significantly different (*p* < 0.05).

### 3.5 Microbial count

Microbial safety is important in determining the quality and shelf life of fresh‐cut fruits. Since minimal processing exposes tissues of fresh‐cut fruit to microbial contamination, the ability of fresh‐cut product to maintain microbial stability during storage is considered crucial. In this study, the effects of chitosan and MA packaging on total bacterial and fungal count were examined. From the result for bacterial count (Table 2), the initial bacterial count was 3.29 CFU/g. Chitosan‐coated samples showed decrease in bacterial count for the first 9 days before a gradual increase in bacterial growth at 12 days of storage. After the storage period, 2% chitosan argon‐based MA packaged samples had the least bacterial count of 2.12 CFU/g, a value lower than the initial count, while uncoated fresh‐cut samples had higher bacterial count ranging from 3.67 to 5.19 CFU/g. Also from the result, it can be seen that nitrogen and argon MA packaging further inhibited rate of bacterial proliferation.

**Table 2:** Total bacteria count (CFU/g) of fresh‐cut cucumber stored at 5°C for 12 days

Bacterial count (log CFU/g)	Storage period (days)				
Treatment	0	3	6	9	12
0CC	3.29 ± 0.04^a^	3.51 ± 0.39^c^	3.71 ± 0.08^e^	4.20 ± 0.08^e^	5.19 ± 0.13^d^
0CN	3.29 ± 0.04^a^	3.31 ± 0.24^c^	3.59 ± 0.10^e^	3.68 ± 0.55^de^	3.81 ± 0.13^c^
0CA	3.29 ± 0.04^a^	2.98 ± 0.04^bc^	3.07 ± 0.08^e^	3.28 ± 0.07^c^	3.67 ± 0.05^bc^
1CC	3.29 ± 0.04^a^	2.76 ± 0.50^bc^	2.85 ± 0.05^d^	3.34 ± 0.04^d^	3.12 ± 0.37^b^
1CN	3.29 ± 0.04^a^	1.42 ± 0.10^ab^	1.99 ± 0.06^bc^	2.74 ± 0.08^c^	2.81 ± 0.36^ab^
1CA	3.29 ± 0.04^a^	1.26 ± 0.80^a^	1.68 ± 0.06^b^	2.54 ± 0.02^b^	2.35 ± 0.00^ab^
2CC	3.29 ± 0.04^a^	2.13 ± 0.83^bc^	2.43 ± 0.16^d^	2.62 ± 0.17^bc^	3.02 ± 0.17^b^
2CN	3.29 ± 0.04^a^	1.83 ± 0.65^b^	2.20 ± 0.29^c^	2.59 ± 0.21^bc^	2.59 ± 0.06^ab^
2CA	3.29 ± 0.04^a^	1.00 ± 0.00^a^	1.42 ± 0.17^a^	1.49 ± 0.78^a^	2.12 ± 0.34^a^

*Note*

Where 0C, 1C, and 2C represents 0%, 1%, and 2% chitosan concentration respectively, and C, N, and A represents control (air‐based), N (nitrogen‐based), and A (argon‐based) MAP, respectively. Values in the same column with different superscripts are significantly different (*p* < 0.05).

For fungal count, chitosan coating and MA packaging effectively reduced fungal count for the first 3 days of storage (Table 3). However, increase in fungal count was observed after 6 days of storage for all treatments. As shown in the result, it can be said that chitosan effectiveness in reducing fungal growth over a long period of storage time may not be adequate. Uncoated and air‐based MA packaged fresh‐cut had consistently high growth rate throughout the storage period and a final fungal count of 5.87 CFU/g. About 2% chitosan‐coated nitrogen‐ and argon‐based MA packaged fresh‐cut cucumber had considerably low fungal counts with values of 2.91 and 2.97 CFU/g, respectively, after storage period. Chitosan has been reported to have bactericidal and fungicidal properties, which makes its effective in inhibiting growth of pathogenic bacteria and fungi (Devlieghere, Vermeulen, & Debevere, 2004; Dutta, Tripathi, Mehrotra, & Dutta, 2009; Helander, Nurmiaho‐Lassila, Ahvenainen, Rhoades, & Roller, 2001; Li, Wang, Chen, Huangfu, & Xie, 2008; Waewthongrak, Pisuchpen, & Leelasuphakul, 2015). Also, successful applications of MAP in whole and fresh‐cut fruit and vegetables have been extensively reported in literature (Farber et al., 2003; Sandhya, 2010). The significant ability of nitrogen‐ and argon‐based MA packaging used in this study to inhibit spoilage organisms may be due to their non‐selective antimicrobial effect of high carbon dioxide (Farber et al., 2003).Therefore, it can be concluded from the result that chitosan coating and nitrogen/argon‐based MA packing were very effective in inhibiting bacterial and fungal growth in fresh‐cut cucumber.

**Table 3:** Fungal count (CFU/g) of fresh‐cut cucumber stored at 5°C for 12 days

Fungal count (log CFU/g)	Storage period (days)				
Treatment	0	3	6	9	12
0CC	2.06 ± 0.23^a^	3.75 ± 0.46^b^	3.83 ± 0.02^f^	4.43 ± 0.03^f^	5.87 ± 0.02^f^
0CN	2.06 ± 0.23^a^	1.55 ± 0.35^a^	3.67 ± 0.07^ef^	3.78 ± 0.10^de^	4.55 ± 0.12^d^
0CA	2.06 ± 0.23^a^	1.35 ± 0.00^a^	3.57 ± 0.17^de^	3.53 ± 0.13^d^	4.53 ± 0.13^d^
1CC	2.06 ± 0.23^a^	1.66 ± 0.55^a^	3.62 ± 0.12^de^	4.08 ± 0.03^e^	4.85 ± 0.02^e^
1CN	2.06 ± 0.23^a^	1.45 ± 0.17^a^	3.43 ± 0.05 ^cd^	3.83 ± 0.08^de^	4.06 ± 0.11^c^
1CA	2.06 ± 0.23^a^	1.39 ± 0.08^a^	3.33 ± 0.04^c^	3.16 ± 0.18^c^	3.51 ± 0.22^b^
2CC	2.06 ± 0.23^a^	1.68 ± 0.55^a^	2.31 ± 0.09^b^	3.16 ± 0.05^c^	3.75 ± 0.01^b^
2CN	2.06 ± 0.23^a^	1.52 ± 0.70^a^	2.17 ± 0.11^b^	2.67 ± 0.17^b^	2.91 ± 0.24^a^
2CA	2.06 ± 0.23^a^	1.42 ± 0.20^a^	1.95 ± 0.16^a^	2.28 ± 0.40^a^	2.97 ± 0.25^a^

*Note*

Where 0C, 1C, and 2C represents 0%, 1%, and 2% chitosan concentration respectively, and C, N, and A represents control (air‐based), N (nitrogen‐based), and A (argon‐based) MAP, respectively. Values in the same column with different superscripts are significantly different (*p* < 0.05).

## 4 CONCLUSION

Fresh‐cut cucumber requires quality preservation method sufficient to maintain quality and replenish tissue integrity after minimal processing. The results indicated that chitosan coating to some extent preserved quality of fresh‐cut cucumber, but its effectiveness alone may not be sufficient over long storage period. Packaging in natural air supports rapid tissue respiration and negatively affects quality and promotes senescence in fresh‐cut cucumber. The use of active packaging films can also be suggested for further studies to create a modified atmosphere in packaged fresh‐cut cucumber by its selective gaseous permeability. In general, the combination of chitosan coating with argon‐based MA packaging best preserved quality and prolonged the shelf life of fresh‐cut cucumber throughout 12 days storage. Therefore, it can inferred that the combination of chitosan treatment and argon‐based MA packing have potential application in the food industry to preserve the overall quality and extend the shelf life of the fresh‐cut cucumber.

## CONFLICT OF INTEREST

The authors of this paper declare no conflict of interest.

## ETHICAL REVIEW

This study does not involve any human or animal testing.

## References

[fsn3937-bib-0001] Ali, A. , Noh, N. M. , & Mustafa, M. A. (2015). Antimicrobial activity of chitosan enriched with lemongrass oil against anthracnose of bell pepper. Food Packaging and Shelf Life, 3, 56–61. 10.1016/j.fpsl.2014.10.003

[fsn3937-bib-0002] Amanatidou, A. , Slump, R. , Gorris, L. , & Smid, E. (2000). High oxygen and high carbon dioxide modified atmospheres for shelf‐life extension of minimally processed carrots. Journal of Food Science, 65(1), 61–66. 10.1111/j.1365-2621.2000.tb15956.x

[fsn3937-bib-0003] Artés, F. , & Allende, A. (2005). Processing lines and alternative preservation techniques to prolong the shelf‐life of minimally fresh processed leafy vegetables. European Journal of Horticultural Science, 70, 231–245.

[fsn3937-bib-0004] Bai, J. H. , Saftner, R. , Watada, A. , & Lee, Y. (2001). Modified atmosphere maintains quality of fresh‐cut cantaloupe (*Cucumis melo* L.). Journal of Food Science, 66(8), 1207–1211. 10.1111/j.1365-2621.2001.tb16106.x

[fsn3937-bib-0005] Bal, E. (2018). Postharvest application of chitosan and low temperature storage affect respiration rate and quality of plum fruits. Journal of Animal Science and Technology, 15, 1219–1230.

[fsn3937-bib-0006] Barrett, D. M. , Beaulieu, J. C. , & Shewfelt, R. (2010). Color, flavor, texture, and nutritional quality of fresh‐cut fruits and vegetables: Desirable levels, instrumental and sensory measurement, and the effects of processing. Critical Reviews in Food Science and Nutrition, 50(5), 369–389. 10.1080/10408391003626322 20373184

[fsn3937-bib-0007] Bastrash, S. , Makhlouf, J. , Castaigne, F. , & Willemot, C. (1993). Optimal controlled atmosphere conditions for storage of broccoli florets. Journal of Food Science, 58(2), 338–341. 10.1111/j.1365-2621.1993.tb04270.x

[fsn3937-bib-0008] Bhande, S. , Ravindra, M. , & Goswami, T. (2008). Respiration rate of banana fruit under aerobic conditions at different storage temperatures. Journal of Food Engineering, 87(1), 116–123. 10.1016/j.jfoodeng.2007.11.019

[fsn3937-bib-0009] Brasil, I. , Gomes, C. , Puerta‐Gomez, A. , Castell‐Perez, M. , & Moreira, R. (2012). Polysaccharide‐based multilayered antimicrobial edible coating enhances quality of fresh‐cut papaya. LWT‐Food Science and Technology, 47(1), 39–45. 10.1016/j.lwt.2012.01.005

[fsn3937-bib-0010] Brecht, J. K. (1995). Physiology of lightly processed fruits and vegetables. HortScience, 30(1), 18–22.

[fsn3937-bib-0011] Chien, P.‐J. , Sheu, F. , & Yang, F.‐H. (2007). Effects of edible chitosan coating on quality and shelf life of sliced mango fruit. Journal of Food Engineering, 78(1), 225–229. 10.1016/j.jfoodeng.2005.09.022

[fsn3937-bib-0012] Coppens d'Eeckenbrugge, G. , Leal, F. , Bartholomew, D. , Paull, R. , & Rohrbach, K. (2003). The pineapple: Botany, production and uses (pp. 13–32). Oxon, UK: CABI.

[fsn3937-bib-0700] Day, B. P. F. (1996). High oxygen modified atmosphere packaging for fresh prepared produce. Postharvest News and Information, 7, 31N–34N.

[fsn3937-bib-0701] Day, B. (1998). Novel MAP: A brand new approach. Food Manufacture, 73(11), 22–24.

[fsn3937-bib-0013] Day, B. (2007). Modified atmosphere packaging (MAP) a global perspective on new developments. 40th AIFST Convention. Melbourne *, June, 25*, 2007.

[fsn3937-bib-0014] Devlieghere, F. , Vermeulen, A. , & Debevere, J. (2004). Chitosan: Antimicrobial activity, interactions with food components and applicability as a coating on fruit and vegetables. Food Microbiology, 21(6), 703–714. 10.1016/j.fm.2004.02.008

[fsn3937-bib-0015] Dutta, P. , Tripathi, S. , Mehrotra, G. , & Dutta, J. (2009). Perspectives for chitosan based antimicrobial films in food applications. Food Chemistry, 114(4), 1173–1182. 10.1016/j.foodchem.2008.11.047

[fsn3937-bib-0016] Farber, J. , Harris, L. , Parish, M. , Beuchat, L. , Suslow, T. , Gorney, J. , & Busta, F. (2003). Microbiological safety of controlled and modified atmosphere packaging of fresh and fresh‐cut produce. Comprehensive Reviews in Food Science and Food Safety, 2(s1), 142–160. 10.1111/j.1541-4337.2003.tb00032.x

[fsn3937-bib-0017] Ghidelli, C. , Mateos, M. , Rojas‐Argudo, C. , & Pérez‐Gago, M. B. (2014). Extending the shelf life of fresh‐cut eggplant with a soy protein–cysteine based edible coating and modified atmosphere packaging. Postharvest Biology and Technology, 95, 81–87. 10.1016/j.postharvbio.2014.04.007

[fsn3937-bib-0018] Gil, M. I. , & Allende, A. (2012). Minimal processing In Gómez‐López V. M. (Ed.), Decontamination of fresh and minimally processed produce (pp. 105–120). Hoboken, NJ: John Wiley & Sons Inc 10.1002/9781118229187

[fsn3937-bib-0019] Greenwood, N. N. , & Earnshaw, A. (2012). Chemistry of the elements. Amsterdam, The Netherlands: Elsevier.

[fsn3937-bib-0020] Helander, I. , Nurmiaho‐Lassila, E.‐L. , Ahvenainen, R. , Rhoades, J. , & Roller, S. (2001). Chitosan disrupts the barrier properties of the outer membrane of Gram‐negative bacteria. International Journal of Food Microbiology, 71(2–3), 235–244. 10.1016/S0168-1605(01)00609-2 11789941

[fsn3937-bib-0021] IFPA (2004). The international fresh‐cut industry. Alexandria, VA: International Fresh‐Cut Produce Association.

[fsn3937-bib-0022] Jamie, P. , & Saltveit, M. E. (2002). Postharvest changes in broccoli and lettuce during storage in argon, helium, and nitrogen atmospheres containing 2% oxygen. Postharvest Biology and Technology, 26(1), 113–116. 10.1016/S0925-5214(02)00006-6

[fsn3937-bib-0023] Jianglian, D. , & Shaoying, Z. (2013). Application of chitosan based coating in fruit and vegetable preservation: A review. Journal of Food Processing and Technology, 4(5), 227.

[fsn3937-bib-0024] Kanellis, A. , Morris, L. , & Saltveit Jr, M. (1986). Effect of stage of development on postharvest behavior of cucumber fruit. HortScience (USA), 21(1986), 1165–1167.

[fsn3937-bib-0025] Kochhar, V. , & Kumar, S. (2015). Effect of different pre‐cooling methods on the quality and shelf life of broccoli. Food Processing and Technology, 6(3), 1–7.

[fsn3937-bib-0026] Krebbers, B. , Matser, A. , Koets, M. , & Van den Berg, R. (2002). Quality and storage‐stability of high‐pressure preserved green beans. Journal of Food Engineering, 54(1), 27–33. 10.1016/S0260-8774(01)00182-0

[fsn3937-bib-0027] Li, B. , Wang, X. , Chen, R. , Huangfu, W. , & Xie, G. (2008). Antibacterial activity of chitosan solution against *Xanthomonas* pathogenic bacteria isolated from *Euphorbia pulcherrima* . Carbohydrate Polymers, 72(2), 287–292. 10.1016/j.carbpol.2007.08.012

[fsn3937-bib-0028] Linga, P. , Kumar, R. , & Englezos, P. (2007). Gas hydrate formation from hydrogen/carbon dioxide and nitrogen/carbon dioxide gas mixtures. Chemical Engineering Science, 62(16), 4268–4276. 10.1016/j.ces.2007.04.033

[fsn3937-bib-0029] Liu, C. , Liu, W. , Lu, X. , Ma, F. , Chen, W. , Yang, J. , & Zheng, L. (2014). Application of multispectral imaging to determine quality attributes and ripeness stage in strawberry fruit. PLoS One, 9(2), e87818 10.1371/journal.pone.0087818 24505317 PMC3913704

[fsn3937-bib-0030] Martiñon, M. E. , Moreira, R. G. , Castell‐Perez, M. E. , & Gomes, C. (2014). Development of a multilayered antimicrobial edible coating for shelf‐life extension of fresh‐cut cantaloupe (*Cucumis melo* L.) stored at 4 C. LWT‐Food Science and Technology, 56(2), 341–350.

[fsn3937-bib-0031] Meng, X. , Zhang, M. , Zhan, Z. , & Adhikari, B. (2014). Changes in quality characteristics of fresh‐cut cucumbers as affected by pressurized argon treatment. Food and Bioprocess Technology, 7(3), 693–701. 10.1007/s11947-013-1092-x

[fsn3937-bib-0032] Min, S. , & Krochta, J. M. (2005). Inhibition of penicillium commune by edible whey protein films incorporating lactoferrin, lacto‐ferrin hydrolysate, and lactoperoxidase systems. Journal of Food Science, 70(2), M87–M94. 10.1111/j.1365-2621.2005.tb07108.x

[fsn3937-bib-0033] Moalemiyan, M. , & Ramaswamy, H. (2012). Quality retention and shelf‐life extension in mediterranean cucumbers coated with a pectin‐based film. Journal of Food Research, 1(3), 159 10.5539/jfr.v1n3p159

[fsn3937-bib-0034] Olivas, G. , & Barbosa‐Cánovas, G. (2005). Edible coatings for fresh‐cut fruits. Critical Reviews in Food Science and Nutrition, 45(7–8), 657–670. 10.1080/10408690490911837 16371333

[fsn3937-bib-0035] Oms‐Oliu, G. , Soliva‐Fortuny, R. , & Martín‐Belloso, O. (2007). Effect of ripeness on the shelf‐life of fresh‐cut melon preserved by modified atmosphere packaging. European Food Research and Technology, 225(3–4), 301–311. 10.1007/s00217-006-0415-9

[fsn3937-bib-0036] Oms‐Oliu, G. , Soliva‐Fortuny, R. , & Martín‐Belloso, O. (2008). Using polysaccharide‐based edible coatings to enhance quality and antioxidant properties of fresh‐cut melon. LWT‐Food Science and Technology, 41(10), 1862–1870. 10.1016/j.lwt.2008.01.007

[fsn3937-bib-0037] Pavlath, A. , & Orts, W. (2009). Edible film and coatings for food application. Chapter 1: Edible films and coating: Why, what and how. New York, NY: Springer.

[fsn3937-bib-0038] Rico, D. , Martin‐Diana, A. B. , Barat, J. , & Barry‐Ryan, C. (2007). Extending and measuring the quality of fresh‐cut fruit and vegetables: A review. Trends in Food Science and Technology, 18(7), 373–386. 10.1016/j.tifs.2007.03.011

[fsn3937-bib-0039] Rojas‐Graü, M. , Tapia, M. , Rodríguez, F. , Carmona, A. , & Martin‐Belloso, O. (2007). Alginate and gellan‐based edible coatings as carriers of antibrowning agents applied on fresh‐cut Fuji apples. Food Hydrocolloids, 21(1), 118–127. 10.1016/j.foodhyd.2006.03.001

[fsn3937-bib-0040] Saltveit, M. E. (2001). A summary of CA requirements and recommendations for vegetables. Acta Horticulturae, 600, 723–727. VIII International Controlled Atmosphere Research Conference.

[fsn3937-bib-0702] Sandhya (2010). Modified atmosphere packaging of fresh produce: Current status and future needs. LWT‐Food Science and Technology, 43(3), 381–392. 10.1016/j.lwt.2009.05.018

[fsn3937-bib-0041] Sidhu, J. S. , & Al‐Zenki, S. F. (2005). Fruits: Horticultural and functional properties In Hui Y. H. (Ed.), Handbook of food science, technology, and engineering‐4 volume set, Chap. 24 (pp. 1–28). Boca Raton, FL: Taylor and Francis Group.

[fsn3937-bib-0703] Singh, V. , Hedayetullah, M. , Zaman, P. , & Meher, J. (2014). Postharvest technology of fruits and vegetables: An overview. Journal of Postharvest Technology, 2(2), 124–135.

[fsn3937-bib-0042] Spencer, K. (1995). The use of argon and other noble gases for the MAP of foods. Paper presented at the International conference on MAP and related technologies.

[fsn3937-bib-0043] Spencer, K. C. (2005). Modified atmosphere packaging of ready‐to‐eat foods In Han J. H. (Ed.), Innovations in food packaging (pp. 185–203). London, UK: Elsevier.

[fsn3937-bib-0044] Waewthongrak, W. , Pisuchpen, S. , & Leelasuphakul, W. (2015). Effect of *Bacillus subtilis* and chitosan applications on green mold (*Penicilium digitatum* Sacc.) decay in citrus fruit. Postharvest Biology and Technology, 99, 44–49. 10.1016/j.postharvbio.2014.07.016

[fsn3937-bib-0045] Wong, D. W. , Tillin, S. J. , Hudson, J. S. , & Pavlath, A. E. (1994). Gas exchange in cut apples with bilayer coatings. Journal of Agricultural and Food Chemistry, 42(10), 2278–2285. 10.1021/jf00046a037

[fsn3937-bib-0046] Wu, Z. , Zhang, M. , & Wang, S. (2012). Effects of high pressure argon treatments on the quality of fresh‐cut apples at cold storage. Food Control, 23(1), 120–127. 10.1016/j.foodcont.2011.06.021

[fsn3937-bib-0047] Yang, L. , Tulk, C. , Klug, D. , Chakoumakos, B. , Ehm, L. , Molaison, J. , & Simonson, J. (2010). Guest disorder and high pressure behavior of argon hydrates. Chemical Physics Letters, 485(1–3), 104–109. 10.1016/j.cplett.2009.12.024

[fsn3937-bib-0048] Zhang, D. , Quantick, P. C. , Grigor, J. M. , Wiktorowicz, R. , & Irven, J. (2001). A comparative study of effects of nitrogen and argon on tyrosinase and malic dehydrogenase activities. Food Chemistry, 72(1), 45–49. 10.1016/S0308-8146(00)00201-6

[fsn3937-bib-0049] Zhang, M. , Zhan, Z. , Wang, S. , & Tang, J. (2008). Extending the shelf‐life of asparagus spears with a compressed mix of argon and xenon gases. LWT‐Food Science and Technology, 41(4), 686–691. 10.1016/j.lwt.2007.04.011

